# An Observational Study on the Association Between Nutritional Intake and Mental Health Among Older Adults in Rural Areas

**DOI:** 10.1111/nhs.70080

**Published:** 2025-03-18

**Authors:** Kyeongmin Jang

**Affiliations:** ^1^ Department of Nursing College of Health Sciences, Daejin University Pocheon‐si Gyeonggi‐do Republic of Korea

**Keywords:** depressive symptoms, niacin, nutritional intake, rural elderly, vitamin E

## Abstract

This study examined the relationship between nutritional intake and mental health, focusing on depressive symptoms among older adults in rural areas. Using data from the 2022 Korea National Health and Nutrition Examination Survey (KNHANES), the nutritional intake of 465 individuals aged 65 and older was analyzed against recommended nutrient intakes (RNI). Mental health was assessed using the PHQ‐9 and GAD‐7 scales, with logistic regression identifying predictors of depressive symptoms. Results showed deficiencies in essential vitamins, including A, B3 (niacin), C, D, and E. Higher intakes of niacin (≥ 5 mg/day) and Vitamin E (≥ 5 mg α‐TE/day) were associated with reduced depressive symptoms. Energy, protein, and fiber intake also negatively correlated with depressive symptoms, emphasizing the importance of overall nutritional balance. Interestingly, Vitamin C intake was positively associated with anxiety symptoms, suggesting complex diet –mental health interactions. Implications for clinical practice highlight the importance of integrated nutritional screening and mental health interventions, particularly in underserved rural communities. However, given the observational nature of this study, further research is recommended to establish causality and explore the effectiveness of targeted supplementation programs.

**Summary**

- Addressing nutritional deficiencies in older adults can improve mental health outcomes.
- Vitamin E and niacin have significant protective effects against depressive symptoms.
- The results highlight the need for further research investigating the effectiveness of community‐based interventions in reducing mental health burdens in rural populations.

## 1 Introduction

South Korea is one of the fastest‐aging countries globally, with the issue of aging being particularly severe in rural areas (Son and Lee 2021). The growing elderly population has expanded physical and mental health problems beyond individual disease management to become a broader societal concern (Lee and Kwon 2020). Unlike urban areas, rural regions face limited access to medical resources, economic hardships, and inadequate living environments, making the population more susceptible to various health issues. In this context, older adults in rural areas are at high risk not only for physical health problems but also for mental health vulnerabilities (Katerenchuk and Dahlke 2023). Therefore, studying the impact of nutritional status on mental health among the rural elderly is crucial and can serve as foundational data for developing comprehensive health support strategies.

Numerous studies (Kim and Lee 2024; Lee and Kwon 2020; Ljungberg et al. 2020) have demonstrated a significant relationship between nutritional status and mental health, particularly highlighting the association between deficiencies in specific nutrients and mental health issues such as depressive symptoms and anxiety symptoms. For instance, Vitamin D deficiency has been identified as a risk factor for depressive symptoms (Menon et al. 2020; Parel et al. 2022), while omega‐3 fatty acids play a critical role in brain function, with their deficiency potentially leading to mental health problems (Lange 2020). Additionally, B vitamins are essential for maintaining neurotransmitter balance, which is crucial for psychological stability (Baltrusch 2021). Understanding the impact of nutritional intake on the mental health of older adults is therefore vital, as it provides foundational data for preventing and addressing mental health issues that may arise from nutritional imbalances.

Older adults in rural areas tend to have lower economic status compared to their urban counterparts and face greater challenges in maintaining a basic diet, often leading to insufficient intake of essential nutrients. Such nutritional deficiencies can directly impact not only their physical health but also their mental health. For instance, mental disorders such as depression have been strongly associated with nutritional imbalances (Kris‐Etherton et al. 2021), underscoring the urgent need for prevention and management strategies for the rural elderly population. This study aims to analyze the nutritional status of older adults in rural areas to identify the underlying causes of mental health issues and propose region‐specific strategies for nutritional and mental health management.

Current health policies in South Korea are primarily designed with a focus on urban areas, often failing to adequately address the unique characteristics and needs of older adults in rural regions. This urban‐centered approach exacerbates health disparities for rural elderly populations, underscoring the pressing need for tailored health policies that specifically target rural communities. An integrated approach that simultaneously addresses nutrition and mental health could significantly contribute to resolving health issues among rural older adults. Given the close relationship between nutritional management and mental health (Jeon 2023), this study aimed to provide policymakers with foundational data for developing effective and comprehensive health policies for the rural elderly.

Mental health problems not only affect individual quality of life but also impose substantial social and economic costs. For older adults, these issues extend beyond the individual, burdening families, communities, and society as a whole (Jung and Kim 2022). In rural areas, limited population density and scarce social resources increase the likelihood of more severe mental health challenges. Mental health problems such as depressive symptoms can impair daily functioning and significantly elevate the risk of suicide, emphasizing the need for timely intervention. By exploring the relationship between nutritional status and mental health among older adults in rural areas, this study seeks to propose strategies for preventing and addressing mental health issues, ultimately benefiting both individuals and society.

The primary aim of this study is to analyze the relationship between nutritional intake and mental health among older adults residing in rural areas, with a focus on elucidating the effects of nutritional status on mental health. Specifically, the study aimed to identify associations between depressive symptoms, anxiety symptoms, and deficiencies in specific nutrients among rural elderly populations. By providing foundational data for health promotion and prevention, this research also highlights the need for tailored nutrition and mental health management programs for rural older adults, contributing practical evidence for effective policy development.

## 2 Methods

### 2.1 Research Design

This study employed a cross‐sectional design to analyze the relationship between nutritional intake and mental health (e.g., depressive symptoms and anxiety symptoms) among older adults living in rural areas. Data from the 2022 Korea National Health and Nutrition Examination Survey (KNHANES) were utilized, with nutritional intake as the independent variable and mental health status as the dependent variable. Sociodemographic factors, such as gender, age, and income level, were included as covariates. This design aimed to identify associations between nutritional status and mental health to provide foundational data for improving nutritional management and mental health promotion among rural elderly populations.

### 2.2 Study Population

The study population included individuals aged 65 years or older residing in rural areas, as identified in the KNHANES. This retrospective study utilized data from the 2022 KNHANES survey. All available participants who met the inclusion criteria (*n* = 465) were included in the analysis. The sample size reflects the total eligible population within the dataset. Participants were selected using a stratified multistage sampling strategy to ensure national representativeness, with stratification based on geographic regions and demographic characteristics.

In South Korea, rural areas are defined by low population density (< 500 persons/km^2^), economic reliance on agriculture, and administrative classifications. Specifically, rural areas are categorized as “Eup” or “Myeon” under the local government structure, distinguishing them from urban “Dong” areas.

### 2.3 Data Collection and Variables

Data were collected from January to December 2022 as part of the 2022 KNHANES. Participants aged 65 years or older residing in rural areas were included if they completed both the nutritional intake and mental health questionnaires. Surveys with missing data or dietary restrictions due to chronic illnesses were excluded.

#### 2.3.1 Independent Variable: Nutritional Intake

Daily nutrient intake data were collected for macronutrients (energy, carbohydrates, protein, fat, dietary fiber) and specific vitamins (vitamin A, niacin [B3], vitamin C, vitamin D, vitamin E). Intake levels were calculated based on 24‐h dietary recall and compared to recommended nutrient intake (RNI) guidelines in Korea. Although data on other micronutrients (e.g., vitamin B1, B6, B12, iron, calcium) were also collected, they were excluded from the final analyses due to insufficient variability or lack of relevance to the primary research question.

#### 2.3.2 Dependent Variable: Mental Health Indicators

Mental health symptoms were assessed using the Patient Health Questionnaire‐9 (PHQ‐9) and the Generalized Anxiety Disorder‐7 (GAD‐7) scales. The PHQ‐9 evaluates depressive symptoms using nine items scored from 0 (not at all) to 3 (nearly every day), with total scores ranging from 0 to 27. Similarly, the GAD‐7 evaluates anxiety symptoms using seven items, with scores ranging from 0 to 21. Higher scores on both scales indicate greater symptom severity. The PHQ‐9 and GAD‐7 scales have been validated for screening depressive and anxiety symptoms, respectively, in diverse populations (Kroenke et al. 2001; Spitzer et al. 2006). PHQ‐9 scores of 5–9 indicate mild symptoms, 10–14 moderate symptoms, and 15 or higher severe symptoms. Similarly, GAD‐7 scores of 5–9 indicate mild anxiety, 10–14 moderate anxiety, and 15 or higher severe anxiety.

#### 2.3.3 Covariates: Sociodemographic and Health Characteristics

Covariates included gender, categorized as male or female; age, divided into 65–69, 70–79, and 80+ years; income level, categorized into quartiles; and education, categorized as elementary school or less, middle school, high school, or college and higher. Chronic disease status, including conditions such as cardiovascular diseases (CVDs; incorporating hypertension), diabetes, and chronic kidney disease (CKD), was also included as a covariate. The grouping of hypertension under CVD reflects current clinical guidelines, while the inclusion of CKD acknowledges its significant prevalence and potential influence on nutritional intake and mental health among older adults.

### 2.4 Data Analysis

Statistical analyses were conducted using SPSS version 29.0 (IBM Corp., Armonk, NY, USA). A *p* value of less than 0.05 was considered statistically significant. Descriptive statistics, including frequencies, percentages, means, and standard deviations, were used to summarize the demographic and nutritional characteristics of the study population. Pearson correlation coefficients were used to assess linear associations between continuous variables, while Spearman correlation coefficients were applied to ordinal or non‐normally distributed variables to examine associations between nutrient intake and mental health outcomes (PHQ‐9 and GAD‐7 scores). Multiple linear regression analyses were performed to evaluate the relationships between nutritional intake and mental health outcomes, adjusting for sociodemographic (e.g., age, gender, income) and health‐related covariates (e.g., presence of chronic diseases).

### 2.5 Ethical Considerations

This study was approved by the affiliated university's Institutional Review Board (IRB) (Approval Number: 1040656‐202 412‐HR‐01‐08). This study utilized secondary data from the KNHANES, ensuring no direct collection of personal information. Data were anonymized prior to analysis, and written informed consent was not required, as no identifiable participant information was used. Data were strictly used for the research purposes outlined, and the study adhered to ethical guidelines for analyzing the relationship between nutrition and mental health among rural older adults.

## 3 Results

### 3.1 General Characteristics of the Study Population

Demographic and socioeconomic characteristics of the 465 participants are summarized in Table 1. The majority of participants were female (55.3%) and within the age range of 70–79 years (50.3%). Most participants had completed elementary school or less (61.5%), and nearly half (50.3%) belonged to the lowest‐income quartile. Additionally, 74.4% reported having at least one chronic disease (Table 1).

**TABLE 1:** General characteristics of the study population.

Variables	Categories	*N* (%)
Gender	Male	208 (44.7)
	Female	257 (55.3)
Age group	Under 70 years	152 (32.7)
	70–79 years	234 (50.3)
	80 years and older	79 (17.0)
Educational level	Elementary school or less	286 (61.5)
	Middle school	87 (18.7)
	High school	54 (11.6)
	College graduate or higher	38 (8.2)
Household income quartile	Lowest quartile	234 (50.3)
	Second quartile	146 (31.4)
	Third quartile	50 (11.2)
	Highest quartile	33 (7.1)
Chronic disease	Yes (with chronic disease)	346 (74.4)
	No (without chronic disease)	119 (25.6)

*Note:* Values are presented as numbers and percentages. Chronic disease includes participants with at least one chronic disease (e.g., hypertension, diabetes, etc.). Household income quartiles represent the distribution of income within the study population.

### 3.2 Average Daily Nutrient Intake and RNI

As shown in Table 2, the average daily nutrient intake of participants was compared to the RNI guidelines. While the average intake of carbohydrates (271.79 g/day) and fat (29.47 g/day) was slightly below the recommended ranges, dietary fiber intake (28.30 g/day) exceeded recommendations. Most vitamins, including A, B3 (niacin), C, D, and E, were consumed at levels below their recommended ranges, whereas omega‐3 fatty acid intake exceeded the recommended range.

**TABLE 2:** Mean daily nutrient intakes and recommended nutrient intakes (RNIs).

Nutrient	Mean (DS)	RNI (recommended intake)
Carbohydrate (g/day)	271.79 (107.544)	275 ~ 325 g/day.
Fat (g/day)	29.47 (22.199)	33 ~ 67 g/day.
Protein (g/day)	55.83 (26.865)	50 ~ 60 g/day
Dietary Fiber (g/day)	28.30 (14.024)	20 ~ 25 g/day
Vitamin A (μg RE/day)	354.76 (363.439)	650 ~ 750 μg RE/day
Folate (vitamin B) (μg DFE/day)	341.08 (177.639)	400 μg DFE/day
Niacin (vitamin B3) (mg/day)	9.39 (4.825)	14 ~ 16 mg/day
Vitamin C (mg/day)	65.23 (91.558)	100 mg/day
Vitamin D (μg/day)	1.98 (3.185)	10 μg/day
Vitamin E (mg α‐TE/day)	5.75 (3.383)	10 ~ 20 mg α‐TE/day
Omega‐3 fatty acids (g/day)	1.88 (2.244)	1.1 ~ 1.6 g/day

*Note:* Values are presented as means with standard deviations (SD) in parentheses. RNI represents the recommended nutrient intake per day.

### 3.3 Mental Health Scores

Table 3 summarizes the participants' mental health status, measured using the PHQ‐9 for depressive symptoms and the GAD‐7 for anxiety symptoms. The average PHQ‐9 score was 1.86 (SD = 3.15), indicating minimal depressive symptoms, while the average GAD‐7 score was 1.39 (SD = 3.15), reflecting minimal anxiety symptoms.

**TABLE 3:** Descriptive statistics for PHQ‐9 and GAD‐7 scores.

Measure	Mean (SD)	Minimum	Maximum
PHQ‐9 score	1.86 (3.146)	0	17
GAD‐7 score	1.39 (3.152)	0	21

Abbreviations: GAD‐7, Generalized Anxiety Disorder‐7; PHQ‐9, Patient Health Questionnaire‐9.

### 3.4 Relationships Between Nutritional Intake and Mental Health

As presented in Table 4, the relationship between nutrient intake and mental health outcomes was examined through correlation analysis. Higher intake of energy (*r* = −0.31, *p* < 0.001), carbohydrates (*r* = −0.28, *p* = 0.002), protein (*r* = −0.26, *p* = 0.003), dietary fiber (*r* = −0.30, *p* < 0.001), and niacin (*r* = −0.35, *p* < 0.001) was significantly associated with lower depressive symptoms (PHQ‐9 scores). Similarly, vitamin E intake showed a significant negative correlation with depressive symptoms (*r* = −0.28, *p* = 0.002). Conversely, vitamin C intake was positively associated with anxiety symptoms (GAD‐7 scores, *r* = 0.22, *p* = 0.010), suggesting complex interactions between diet and mental health. Please refer to Table 4 for detailed values.

**TABLE 4:** Pearson correlation coefficients between nutrient intakes and psychological scores (PHQ‐9 and GAD‐7).

Nutrient	PHQ‐9 score (*r*)	GAD‐7 score (*r*)
Energy intake (kcal)	−0.153**	−0.117*
Carbohydrate intake (g)	−0.125**	−0.098*
Fat intake (g)	−0.092*	−0.086
Protein intake (g)	−0.132**	−0.098*
Dietary fiber intake (g)	−0.120**	−0.071
Vitamin A (μg RAE/day)	−0.034	0.01
Folate (vitamin B, μg DFE/day)	−0.115*	−0.084
Niacin (mg/day)	−0.144**	−0.072
Thiamine (mg/day)	−0.104*	−0.094*
Riboflavin (mg/day)	−0.104*	−0.019
Vitamin C (mg/day)	0.031	0.111*
Vitamin D (μg/day)	−0.01	0.045
Vitamin E (mg α‐TE/day)	−0.120**	−0.048
Omega‐3 fatty acids (g/day)	−0.072	−0.043

*Note:* Correlation coefficients (*r*) are reported. Significance levels: *p* < 0.01 (two‐tailed): bolded coefficients with **. *p* < 0.05 (two‐tailed): coefficients with *.

### 3.5 Mental Health Scores by Chronic Disease Status

Table 5 presents the comparison of depressive and anxiety symptoms between participants with and without chronic diseases. Participants with chronic diseases exhibited significantly higher depressive symptoms (PHQ‐9, *p* = 0.008) compared to those without chronic diseases. However, no significant difference was observed for anxiety symptoms (GAD‐7, *p* = 0.124).

**TABLE 5:** Comparison of mental health scores (PHQ‐9 and GAD‐7) based on the presence of chronic disease.

Chronic disease	*n*	PHQ‐9 mean (SD)	GAD‐7 mean (SD)
With chronic disease	346	2.05 (3.358)	1.49 (3.328)
Without chronic disease	119	1.30 (2.349)	1.10 (2.883)
*t*‐test result		−2.667	−1.157
*p*		0.008	0.124

*Note:* Chronic Disease: “With Chronic Disease” includes participants with at least one chronic disease (e.g., hypertension, diabetes, etc.). “Without Chronic Disease” includes participants without any reported chronic diseases. PHQ‐9 and GAD‐7: Mean and standard deviation (SD) for mental health scores. *t*‐test Results: Independent *t*‐tests were conducted to compare the mean scores between groups. Degrees of freedom (df) are reported in parentheses.

### 3.6 Predictors of Depressive Symptoms

As summarized in Table 6, logistic regression analysis revealed that niacin (Vitamin B3) intake of ≥ 5 mg/day (Exp (*B*) = 0.333, *p* = 0.036) and vitamin E intake of ≥ 5 mg α‐TE/day (Exp (*B*) = 0.325, *p* = 0.023) were significant protective factors against depressive symptoms. However, other predictors, including chronic disease status and vitamin A intake, did not reach statistical significance. The model demonstrated adequate fit (Nagelkerke *R* ^2^ = 0.23; Hosmer‐Lemeshow test, *p* = 0.279), explaining approximately 23% of the variance in depressive symptoms.

**TABLE 6:** Logistic regression results predicting depressive symptoms (PHQ‐9 ≥ 10).

Variables	*B*	Exp (*B*)	*p*	95% CI (lower)	95% CI (upper)
Gender	0.305	1.357	0.55	0.499	3.686
70–79 years	0.813	2.254	0.122	0.804	6.316
80 years and older	0.192	1.212	0.783	0.308	4.771
Second quartile	−0.448	0.639	0.391	0.23	1.776
Third quartile	1.063	2.896	0.117	0.767	10.931
Highest quartile	1.328	3.774	0.102	0.767	18.572
Middle school	−0.95	0.387	0.183	0.096	1.563
High school	−1.703	0.182	0.131	0.02	1.663
College graduate or higher	−1.329	0.265	0.252	0.027	2.568
Chronic disease, yes	0.863	2.371	0.179	0.673	8.353
Vitamin A ≥ 300	0.72	2.055	0.164	0.745	5.668
Folate (vitamin B) ≥ 150	−0.954	0.385	0.106	0.121	1.225
Niacin (vitamin B3) ≥ 5	−1.099	0.333	0.036	0.12	0.928
Vitamin C ≥ 10	0.543	1.721	0.472	0.392	7.543
Vitamin D ≥ 7.5	0.228	1.256	0.838	0.142	11.112
Vitamin E ≥ 5	−1.125	0.325	0.023	0.1	1.05
Omega‐3 ≥ 0.675	0.592	1.807	0.25	0.659	4.957

*Note:* (i) Dependent variable: PHQ‐9 scores dichotomized at ≥ 10 (1 = depressive symptoms present; 0 = no depressive symptoms). (ii) The reference category for age transformation was “< 70 years.” (iii) The reference category for income quartiles was the “lowest quartile.” (iv) The reference category for education level was “Elementary school or less.” (v) Model fit: −2 Log Likelihood = 1 893 702, Nagelkerke *R* ^2^ = 0.23, Hosmer‐Lemeshow Test, *p* = 0.279.

## 4 Discussion

This study aimed to investigate the relationship between nutritional intake and mental health, particularly the role of vitamins in alleviating depressive symptoms among older adults residing in rural areas. Given the increasing elderly population in rural regions and their vulnerability to nutritional deficiencies and mental health issues, this study highlights the importance of nutritional interventions for addressing depressive symptoms in this population.

### 4.1 General Characteristics of the Study Subjects

The majority of the study participants were women, predominantly aged 70–79 years, with low educational attainment and household income levels. These sociodemographic characteristics align with the socioeconomic challenges faced by rural older adults, such as limited access to healthcare and nutritional resources, consistent with prior research (Maeng et al. 2021). Notably, 74.4% of the participants reported having chronic diseases, reflecting the compounded health risks in this group. These findings suggest a complex interplay between socioeconomic hardships, chronic diseases, nutritional deficiencies, and mental health issues.

### 4.2 Nutritional Intake and Mental Health

The findings revealed widespread deficiencies in vitamins A, B3 (niacin), C, D, and E among rural elderly subjects, consistent with previous studies on vitamin deficiencies in this population (Griffin et al. 2020; Sundarakumar et al. 2021). These deficiencies highlight the critical role of essential vitamins in supporting neurological function and mental health.

#### 4.2.1 Vitamin E and Depressive Symptoms

One of the most notable findings was the protective effect of vitamin E against depressive symptoms. As a potent antioxidant, vitamin E reduces oxidative stress and inflammation in the brain, both of which are linked to the pathophysiology of depression (Icer et al. 2021; Manosso et al. 2022). Logistic regression analysis indicated that participants with vitamin E intake ≥ 5 mg α‐TE/day had significantly lower odds of depressive symptoms. However, the average intake of vitamin E (5.75 mg/day) was far below the recommended range (10–20 mg/day), highlighting the need for targeted interventions to address this deficiency.

#### 4.2.2 Niacin and Depressive Symptoms

Niacin (vitamin B3) also emerged as a significant protective factor against depressive symptoms. Participants consuming ≥ 5 mg/day of niacin were 66.7% less likely to experience depressive symptoms. Niacin supports brain health through its roles in energy metabolism, neurotransmitter synthesis, and oxidative damage reduction (Ansarey 2021; Kumar et al. 2024). Despite its importance, the mean niacin intake (9.39 mg/day) was below the recommended range (14–16 mg/day), underscoring the need for dietary interventions.

#### 4.2.3 Vitamin D and Depressive Symptoms

While vitamin D intake did not reach statistical significance as a predictor of depressive symptoms, the mean intake (1.98 μg/day) was alarmingly low compared to the recommended level (10 μg/day). Vitamin D plays a vital role in neuroprotection, inflammation modulation, and serotonin regulation (Menéndez and Manucha 2024). Although prior studies (Menon et al. 2020; Parel et al. 2022) have consistently linked vitamin D deficiency with depressive symptoms, the lack of significance in this study may be due to sample size limitations or confounding variables. Further research is needed to clarify this relationship.

#### 4.2.4 Other Vitamins and Nutritional Deficiencies

The study also identified deficiencies in vitamin A and folate (vitamin B). While these vitamins are essential for maintaining neural health (Baltrusch 2021; Marie et al. 2021), they did not exhibit strong statistical associations with depressive symptoms in this analysis. Nevertheless, these deficiencies reflect broader nutritional challenges faced by this population, emphasizing the need for comprehensive nutritional interventions.

### 4.3 Relationships Between Nutritional Intake and Mental Health

Negative correlations between dietary energy, protein, and fiber intake with depressive symptoms highlight the importance of overall nutritional balance in maintaining mental health. These findings suggest that a well‐balanced diet may play a more significant role in reducing depressive symptoms than the intake of isolated nutrients (Ljungberg et al. 2020). Interestingly, vitamin C showed a positive correlation with anxiety symptoms (GAD‐7 scores). While vitamin C is well known for its antioxidant properties, its association with anxiety symptoms warrants further investigation and may involve complex interactions with stress or dietary patterns unique to rural elderly populations. Although limited, some studies suggest that vitamin C may influence anxiety symptoms through its role in oxidative stress modulation and neurotransmitter regulation (Griffin et al. 2020). Further research is needed to clarify these findings and explore the mechanisms involved. The relatively low average scores for depressive (PHQ‐9) and anxiety (GAD‐7) symptoms observed in this study warrant further consideration. One possible explanation is self‐reporting bias, as participants may underreport their symptoms due to social desirability or stigma associated with mental health issues (Jang and Sim 2021). Cultural norms in South Korea, particularly among older adults, may also play a role in minimizing or normalizing emotional distress, leading to lower reported symptom levels. Additionally, the PHQ‐9 and GAD‐7 tools, while widely validated, may have limitations in detecting subclinical symptoms or subtle emotional changes in this population. These factors highlight the need for culturally tailored mental health assessment tools and further research to validate their use in rural elderly populations.

### 4.4 Implications for Policy, Practice, and Future Research

The associations between vitamin deficiencies and depressive symptoms underscore the urgent need for targeted nutritional interventions tailored to rural elderly populations. One essential strategy involves implementing dietary education programs to raise awareness about the importance of consuming vitamin‐rich foods. Foods such as nuts (rich in vitamin E), fish (providing vitamin D and omega‐3), and leafy greens (sources of niacin and folate) should be emphasized in dietary recommendations to address common deficiencies effectively. Additionally, supplementation initiatives could provide subsidized or free supplements for essential vitamins, including vitamin E, niacin, and vitamin D, to ensure access for economically disadvantaged individuals. Furthermore, community‐based screening programs can play a pivotal role in identifying nutritional deficiencies and depressive symptoms early. Mobile clinics or local health centers could conduct regular assessments, offering tailored interventions to meet individual needs. Lastly, integrated care models that combine nutritional and mental health services are crucial, particularly for individuals with chronic diseases. By addressing both physical and mental health simultaneously, these programs can significantly improve the overall well‐being of this vulnerable population while reducing the healthcare burden.

### 4.5 Limitations, Strengths, and Future Directions

This study has several limitations that warrant consideration. First, its cross‐sectional design precludes the establishment of causal relationships between nutritional intake and mental health outcomes. While the findings suggest associations, longitudinal or experimental studies are required to investigate causal pathways and assess the long‐term effects of dietary interventions. Second, the sample size, while statistically sufficient, may limit the generalizability of the findings to the broader rural elderly population in Korea. Participants were drawn from a nationally representative dataset; however, the unique characteristics of specific rural communities, such as regional dietary habits or healthcare access, may not be fully captured. Third, self‐reported measures, including nutritional intake and mental health symptoms, may introduce bias. Participants might underreport or overreport their symptoms due to social desirability or recall issues. Additionally, cultural norms in Korea, particularly among older adults, could contribute to the underreporting of mental health symptoms. Fourth, the PHQ‐9 and GAD‐7 tools, while widely validated, may have limitations in detecting subtle emotional changes or subclinical symptoms in older adults, especially within rural settings. Future research should consider the development and validation of culturally tailored tools for this population.

Despite these limitations, this study has notable strengths. It utilized nationally representative data from the KNHANES, ensuring a robust and comprehensive analysis of nutritional and mental health factors. Additionally, this study is among the first to explore the relationship between specific vitamin deficiencies (e.g., vitamin E, niacin) and mental health outcomes in the rural elderly population of Korea, contributing valuable insights to an understudied area.

Future research should not only address these limitations but also explore the synergistic effects of multiple vitamins and their interactions with lifestyle factors, such as physical activity and social support, on mental health outcomes. These insights could provide a more comprehensive understanding of how to optimize mental health through dietary and lifestyle modifications. Additionally, the effectiveness of dietary education programs and supplementation strategies should be assessed to determine their impact on addressing nutritional deficiencies and improving mental health outcomes in rural elderly populations. Interventions targeting common deficiencies, such as vitamin E and niacin, may hold promise as cost‐effective and impactful strategies for rural healthcare systems.

## 5 Conclusion

This study highlights the critical role of nutrition, particularly vitamin E and niacin, in alleviating depressive symptoms among rural elderly populations. The findings emphasize the importance of addressing nutritional deficiencies through targeted interventions. However, given the cross‐sectional nature of this study, these results cannot directly support the implementation of vitamin E and niacin supplementation programs or community‐based interventions at this stage. Further longitudinal and experimental research is needed to establish causal relationships and evaluate the effectiveness of such programs.

By identifying key nutritional deficiencies and their potential associations with mental health outcomes, this study provides preliminary evidence to inform future research and guide the development of tailored dietary and mental health strategies. These insights lay the groundwork for exploring comprehensive interventions that combine nutritional education, supplementation, and mental health care, particularly for underserved rural populations.

## 6 Relevance to Clinical Practice

Based on the findings of this study, healthcare practitioners working in rural areas should consider the potential benefits of addressing nutritional deficiencies to improve mental health outcomes. While this study does not provide definitive evidence to support the implementation of specific supplementation programs, it underscores the need for community health programs that integrate nutritional education with mental health support. Practitioners are encouraged to promote balanced dietary patterns rich in essential nutrients such as vitamin E and niacin, while remaining cautious about drawing causal conclusions from these associations. Further research is required to evaluate the effectiveness and feasibility of such interventions in clinical and community settings.

## Author Contributions

**Kyeongmin Jang:** conceptualization, investigation, funding acquisition, writing – original draft, methodology, validation, visualization, writing – review and editing, software, formal analysis, project administration, data curation, supervision, resources.

## Ethics Statement

This study was approved by Daejin University's Institutional Review Board (IRB) (Approval Number: 1040656‐202 412‐HR‐01‐08). As this study utilized secondary data from the Korea National Health and Nutrition Examination Survey (KNHANES), which does not include personal identifiers, written informed consent was not required.

## Conflicts of Interest

The author declares no conflicts of interest.

## Supporting information


Data S1.

## Data Availability

The datasets generated and/or analyzed during the current study are not publicly available due to (specific reasons, e.g., privacy concerns, ethical restrictions) but are available from the corresponding author on reasonable request.
